# Effectiveness of a training program in compliance with recommendations for venous lines care

**DOI:** 10.1186/s12879-015-1046-1

**Published:** 2015-07-30

**Authors:** M. J. Pérez-Granda, M. Guembe, C. Rincón, P. Muñoz, E. Bouza

**Affiliations:** Cardiac Surgery Postoperative Care Unit, Hospital General Universitario Gregorio Marañón, Madrid, Spain; Department of Clinical Microbiology and Infectious Diseases, Hospital General Universitario Gregorio Marañón, Doctor Esquerdo, 46; 28007 Madrid, Spain; Medicine Department, School of Medicine, Universidad Complutense de Madrid, Madrid, Spain; CIBER Enfermedades Respiratorias-CIBERES (CB06/06/0058), Madrid, Spain

**Keywords:** Surveillance, Venous lines, ICU, Non-ICU

## Abstract

**Background:**

The impact of training programs on the care and maintenance of venous lines (VL) has been assessed mainly in patients admitted to the intensive care unit (ICU). Data on the impact of such programs in a whole general hospital are scarce. The objective of this study was to assess compliance with VL care after an extensive training program aimed at nurses caring for adult ICU and non-ICU patients.

**Methods:**

We performed 2 point prevalence studies in a general hospital. A specialized nurse visited all hospitalized adult patients, performed a bedside inspection, and reviewed the nursing records for patients with a VL before and after a 1-year training program. The program included an interactive on-line teaching component and distribution of pocket leaflets and posters with recommendations on VL care.

**Results:**

Data recorded for the first and second prevalence studies were as follows: number of patients visited, 753 vs. 682; total number of patients with ≥ 1 VL implanted on the visit day, 653 (86.7 %) vs 585 (85.8 %); catheters considered unnecessary on the study day, 183 (22.9 %) vs 48 (7.1 %) (*p* < 0.001); number of catheters with local clinical evidence of infection on the study day, 18 (2.2 %) vs 12 (1.8 %) (*p* = 0.52); registration of insertion day (42.3 % vs 50.1 %; *p* = 0.003); and registration of day of dressing change (41.2 % vs 49.1 %; *p* = 0.003). Maintenance parameters improved more in non-ICU than in ICU patients.

**Conclusion:**

A multidisciplinary teaching program to improve VL care and compliance with recommendations is effective. Point prevalence studies are easy to carry out and effective at demonstrating increases in compliance, mainly in non-ICU patients.

## Background

Catheter-related infection (CRI) is a major nosocomial infection that is associated with an increase in morbidity, mortality, and hospital stay [1–5].

Data on bedside practice in prevention of infection of central and peripheral venous lines (VL) in a general institution are scarce [6–9]. Information is mainly from ICU patients, and few studies compare catheter care in ICU patients and non-ICU patients [10–16].

We evaluated the efficacy of bedside point prevalence studies to assess compliance with the recommendations provided during a campaign to promote good care of VLs [17].

## Methods

### Setting and patients

The study was performed in a general referral hospital with 1,550 beds and approximately 50,000 admissions/year.

Two point prevalence studies were performed (January 2013 and September 2014). A nurse visited all adult patients (psychiatry and maternity were excluded) who were admitted at the time of the visit and had ≥1 peripheral or central VLs inserted at the time of the visit. Arterial catheters were excluded. The same methodology was used in both studies. After the first study, an educational intervention was implemented. The intervention comprised an interactive on-line training program, distribution of pocket leaflets with recommendations on catheter care, posters in all nursing units, and talks to small groups of nurses during the different shifts.

Our recommendations were the following: use of chlorhexidine with alcohol 2 %, use of connectors (split-septum) in all hubs, daily surveillance, replacement of dressings every 2 days (gauze dressings) and 7 days (transparent dressings), replacement of administration sets every 7 days (including connectors), and removal of unnecessary catheters.

The intervention was performed by a multidisciplinary group of infectious diseases specialists, infection control physicians, and nurses, who were appointed by the Infection Control Committee. We preferred bedside visits because they provide a more direct way of assessing clinical practice and the training program. The point prevalence studies were easy to perform and affordable for an institution with limited resources. The online self-teaching modules were recommended directly to all nurses interviewed and advertised on the hospital web page. The program was not compulsory and was also promoted among physicians. The talks given to small groups of nurses were delivered during all 3 shifts and had an initial pre-established format followed by questions and a discussion. The main barriers to delivering the talks were the need to address staff across 3 shifts and the need to give the talks at the nursing station so that as many all health care workers as possible could be addressed.

### Data collection

The bedside data collected during the study visit included age, sex, ward of admission, and number of VLs.

For each VL, we recorded location of catheter, type of catheter, use of connectors in the different hubs, and the main reason for use of the VL and clinical signs of infection at the catheter site.

The attending nurses were interviewed and a series of data was recorded (date of insertion of each VL, registry of daily surveillance, date of last dressing change, and date of last administration set replacement). The nurses gave their consent to participate in the study.

A discussion was held with the head nurse on the need for a VL for every catheter. We considered the VL to be unnecessary on the study day when the patient was hemodynamically stable, had no indication for IV fluids via that line, and did not require IV medication.

Finally, a line was considered adequate when all the following criteria were fulfilled:

1. Registration of insertion date
2. Registration of dressing change date
3. Registration of administration set replacement date
4. Registration of daily surveillance
5. Connectors locking all lumens and hubs
6. Need for a VL on the study day
7. No evidence of local signs of infection at the catheter entry site

Even when phlebitis or induration was not infectious, we defined clinical evidence of local infection when there was at least 1 of the following manifestations: phlebitis, erythema, induration, redness, or suppuration.

### Ethics

The Ethics Committee of Hospital Gregorio Marañon approved the study. Patients did not have to give their written informed consent because the study was observational (no interventions).

### Statistical analysis

Qualitative variables are expressed with their frequency distribution. The quantitative variables are expressed as the mean and standard deviation (SD). Continuous variables were compared using the *t* test if they were normally distributed and the Kruskal-Wallis test if they were not normally distributed. The chi-square or Fisher exact test was used to compare categorical variables. Quantitative variables were compared using parametric methods (*t* test or analysis of variance).

All statistical tests were 2-tailed. Statistical significance was set at *p* < 0.05 for all the tests. The statistical analysis was performed with SPSS 12.0.

## Results

The data recorded for the first and second study include the following: number of patients visited, 753 vs. 682 (*p* = 0.90); patients with one or more VLs on the study day, 653 (86.7 %) vs 585 (85.8 %) *p* = 0.77; total number of catheters implanted at the time of the visit, 797 vs 678 (*p* = 0.64); central VLs, 144 (18.1 %) vs 122 (18.0 %); and peripheral VLs 653 (81.9 %) vs 556 (82.0 %) (Table 1).

**Table 1:** General data of study 1 and study 2

General data	Study 1	Study 2	p
No (%) of patients visited	753	682	0.90
ICU	52 (6.9)	46 (6.7)	
Non-ICU	701 (93.1)	636 (93.2)	
Mean age in years (SD)	67.6 (17.1)	65.2 (17.7)	<0.001
Sex M/F	429/324	394/288	0.76
No (%) of patients with ≥ 1 catheter inserted	653 (86.7)	585 (85.8)	0.60
Total number of inserted catheters at the time of the visit (%)	797	678	0.64
ICU	104 (13.0)	83 (12.2)	
Non-ICU	693 (87.0)	595 (87.8)	
Type of lines (%)			0.97
No. (%) of central venous lines	144 (18.1)	122 (18.0)	
No. (%) of peripheral venous lines	653 (81.9)	556 (82.0)	
No. (%) of catheters considered unnecessary on the study day	183 (22.9)	48 (7.1)	<0.001
Local clinical evidence of infection, No (%)	18 (2.2)	12 (1.8)	0.52

*ICU* intensive care unit, *non-ICU* non–intensive care unit, *SD*standard deviation, *M* male, *F* female, *CVL* central venous line, *PVL* peripheral venous line

The characteristics of the inserted VLs are summarized in Table 2.

**Table 2:** Characteristics of the catheters

Outcome	Study 1, No. (%)		Study 2, No. (%)		p
	797		678		
	ICU (%)	Non-ICU (%)	ICU (%)	Non-ICU (%)	
Line type, No. (%)	104	693	83	595	0.43
Peripheral	59 (56.7)	594 (85.7)	45 (54.2)	511 (85.9)	
Non-tunneled central venous	42 (40.3)	26 (26.3)	36 (43.4)	23 (3.9)	
Tunneled central venous	2 (1.9)	16 (3.7)	1 (1.2)	14 (2.3)	
Totally implantable	1 (0.9)	51 (7.3)	1 (1.2)	38 (6.4)	
PICC	0 (0.0)	6 (0.8)	0 (0.0)	9 (1.5)	
Insertion site of CVL (PICC excluded)	45	93	38	75	0.66
Jugular	17 (37.7)	7 (7.5)	16 (42.1)	6 (10.3)	
Subclavian	24 (53.3)	82 (88.2)	16 (42.1)	64 (85.3)	
Femoral	4 (8.8)	4 (4.3)	6 (15.8)	3 (4.0)	
Location of peripheral lines (arms), No (%)	59 (9.0)	594 (90.9)	45 (6.6)	508 (74.4)	
Use of catheters^a^	104	693	83	595	<0.001
Medication or fluid	59 (56.7)	501 (72.3)	56 (67.5)	505 (84.9)	
Parenteral nutrition	7 (6.7)	25 (3.6)	6 (7.2)	18 (3.0)	
Hemofiltration	7 (6.7)	15 (2.2)	2 (2.4)	4 (0.7)	

*PICC* peripheral inserted central venous catheter

^a^Excluded unnecessary catheters

On the first study day (before the intervention), 22.9 % of the VLs were unnecessary; after the intervention, the percentage fell to 7.1 % (*p* < 0.001). Clinical evidence of local infection on the study day was detected in 2.2 % vs 1.8 % of catheters (*p* = 0.52). Most of the catheters with signs of local infection were peripheral lines in non-ICU patients.

Data on line maintainance are summarized in Table 3. The registration of the date of insertion of the VLs improved significantly between the first and the second studies (42.3 % vs 50.1 %; *p* < 0.001). Data on the last change of dressing were available in 41.2 % vs 49.1 % (*p* < 0.001), and the set replacement date was registered in 45.4 % vs 43.3 % of cases. We found that the proportion of VLs with split-septum connectors increased from study 1 to study 2 (83.7 % vs. 92.2 %; *p* < 0.001).

**Table 3:** Catheter maintenance

Parameter	Correct		
	Study 1, No. (%)	Study, No. (%)	p
Registration of insertion date	337 (42.3)	339 (50.1)	0.003
Registration of daily surveillance	779 (97.7)	660 (98.2)	0.52
Registration of dressing change date	322 (41.2)	328 (49.1)	0.003
Registration of set replacement date	74 (45.4)	65 (43.3)	0.71
Use of connectors (split-septum) in all hubs	660 (83.7)	615 (92.2)	<0.001

Tables 3 and 4 show the comparison of the main study variables for patients in the ICU and non-ICU setting. The distribution of the workload related to VLs, the use of catheters, and the location and type of catheters were similar between the first and second studies.

**Table 4:** Parameters indicating good infection control between Study 1 and Study 2 and ICU and non-ICU patients

Parameter	ICU (%)	p	Non-ICU (%)	p
	Study 1-Study 2		Study 1-Study 2	
Registration of insertion date	+3.9 %	0.42	+8.9 %	<0.001
	(86.5 %-90.4 %)		(35.6 %-44.5 %)	
Registration of daily surveillance	+3.8 %	0.07	0 %	0.98
	(96.2 %-100 %)		(98.0 %-98.0 %)	
Registration of dressing change date	+8.2 %	0.04	+8.5 %	0.002
	(89.4 %-97.6 %)		(33.0 %-41.5 %)	
Registration of set replacement date	+2.8 %	0.20	+4.0 %	0.78
	(53.8 %-56.6)		(15.3 %-19.3 %)	
Use of connectors (split-septum) in all hubs	+3.9 %	0.41	+8.6 %	<0.001
	(91.3 %-95.2 %)		(81.5 %-90.1 %)	
Necessary lines	+19.0 %	0.002	+15.3 %	<0.001
	(70.2 %-89.2 %)		(78.1 %-93.4 %)	

Table 4 summarizes the results for parameters used to measure improvement in the care of ICU and non-ICU patients.

## Discussion

Our results show that a training program on the care of central and peripheral VLs is effective. Bedside prevalence studies before and after implementation are an adequate instrument for measuring improvements. Although the study was performed both inside and outside the ICU, the program was more effective in non-ICU patients.

Guidelines recommend extending measures for the care of central lines to peripheral catheters both inside and outside the ICU [18]. The reality, however, is that most published studies evaluate the situation of central venous catheters in ICU patients. Data regarding the situation of all the lines in all the patients of an institution are scarce [8–19]. Our study shows that a high proportion of central and peripheral VLs are placed in patients located outside the ICU and that the quality of care was worse for non-ICU patients, as previously demonstrated by Zingg et al. [16].

Mechanisms to control and improve this situation are mainly educational, although the type and nature of the interventions are not frequently described. Our training program included bedside visits, talks to nurses on all shifts, and distribution of handouts with information containing the main recommendations for care of lines.

Objective parameters are necessary to monitor the efficacy of interventions. We found that point prevalence studies based on bedside surveillance are easy to perform and demonstrated the efficacy of the measures implemented.

Our data revealed an improvement in the level of care of VLs after a training program. Some studies show that between 31 % and 83 % of catheters are unnecessary [9–11, 15, 19–22]. However, these studies were performed in critically ill patients and in patients with central VLs. We found that the rate of unnecessary catheters decreased from 22.9 % to 7.1 % (*p* < 0.001) between the first and the second study and that the improvement occurred mainly in non-ICU patients, as reported elsewhere [7, 10–14, 19–22].

The main limitation of our study was that our data cannot be extrapolated to other populations, such as maternity and pediatrics wards.

## Conclusion

A training program for nurses caring for patients with central or peripheral VLs substantially increases compliance with care recommendations, particularly in non-ICU patients. Simple bedside surveillance–based point prevalence studies are an effective means of evaluating the impact of the interventions implemented.

## Abbreviations

ICU: Intensive care unit
VL: Venous lines
CVL: Central venous line
PVL: Peripheral venous line
