# Erratum to: ‘Effectiveness of a training program in compliance with recommendations for venous lines care’

**DOI:** 10.1186/s12879-016-1619-7

**Published:** 2016-06-27

**Authors:** M. J. Pérez-Granda, M. Guembe, C. Rincón, P. Muñoz, E. Bouza

**Affiliations:** Cardiac Surgery Postoperative Care Unit, Hospital General Universitario Gregorio Marañón, Madrid, Spain; Department of Clinical Microbiology and Infectious Diseases, Hospital General Universitario Gregorio Marañón, Madrid, Spain; CIBER Enfermedades Respiratorias-CIBERES (CB06/06/0058, Madrid, Spain; Medicine Department, School of Medicine, Universidad Complutense de Madrid, Madrid, Spain

## Erratum

Unfortunately, the original version of this article [1] contained an error. The financial support was included incorrectly. The correct financial support section can be found below.

## Financial support

M. Guembe is supported by the Miguel Servet Program (ISCIII-MICINN, CP13/00268) from the Health Research Fund (FIS), **of the Carlos III Health Institute (ISCIII), Madrid Spain, partially financed by the by the European Regional Development Fund (FEDER) “A way of making Europe”.** The funders had no role in study design, data collection and analysis, decision to publish, or preparation of the manuscript.
